# Editorial: Gastrointestinal Surgery: Emerging techniques, controversies and state of art

**DOI:** 10.3389/fsurg.2022.1033757

**Published:** 2022-11-02

**Authors:** Francesco Pata, Stefano Rausei, Stefano Scabini, Gianluca Pellino

**Affiliations:** ^1^ General Surgery Unit, Nicola Giannettasio Hospital, Corigliano-Rossano, Italy; ^2^ Sapienza University, Rome, Italy; ^3^ General Surgery Unit, Cittiglio/Angera, ASST Settelaghi, Varese, Italy; ^4^ Oncologic Surgical Unit, Department of Surgery, IRCCS Ospedale Policlinico San Martino, Genoa, Italy; ^5^ Department of Advanced Medical and Surgical Science, Università degli Studi della Campania “Luigi Vanvitelli”, Naples, Italy; ^6^ Colorectal Surgery, Vall d’Hebron University Hospital, Barcelona, Spain

**Keywords:** gastrointestinal surgery, surgical techniques, surgery, artificial intelligence, emerging, new frontiers

**Editorial on the Research Topic** Gastrointestinal Surgery: Emerging techniques, controversies and state of art By Pata F, Rausei S, Scabini S and Pellino G. (2022) Front. Surg. 9: 1033757. doi: 10.3389/fsurg.2022.1033757

“*The abdomen, the chest and the brain will forever be shut from the intrusion of the wise and humane surgeon.*” Sir John Ericksen, 1837

As other medical fields, surgery is currently undergoing a deep transformation (1): on the one hand, emerging technologies such as robotic, artificial intelligence, machine learning, promise to improve standardization and effectiveness of diagnosis and treatment, and on the other hand, humanitarian topics such as disparities in equal access to highest quality surgical care worldwide (2), sustainability (3), gender equality, and patient-reported outcomes are increasingly claimed as priorities in the surgical agenda.

As pointed out by Tekkis et al. in their elegant perspective on 3-D printing technology, the adoption of new technologies is not a passive process: effort is needed from the healthcare systems to understand the advantages against the potential economic limits and the need of integration with traditional technology. The reconceptualization and the adoption of new business models may represent the key to overcome these issues.

The technological revolution impacts also on surgical training. Simulation is becoming a mandatory component for trainees in many universities. Evidence of the best training methods require tailored studies as showed by Sahmand et al. in their study on 3D vs. 2D environment for laparoscopic simulation.

The reduced contribution of high-quality trials and structured research programs in surgery in comparison with other medical fields represents a further issue (4), although observational studies may add robust data when randomized controlled trials are not feasible, unethical, or technically challenging (5).

Tousignant et al. identified the main influencers of surgical efficiency and variability in a 5-year robotic sleeve gastrectomy case series: stomach dissection resulted in the Achilles’ heel in terms of procedure duration. Although external inference may be limited by the single-center design and the limited numbers of the study, it paves the way to larger cohort studies to confirm these findings and to generate plans to reduce times and costs.

Wang et al. in their meta-analysis on early oral feeding after colorectal surgery—including 1,199 patients—confirmed that early oral feeding may represent a safe option, with reduced length of stay and overall complications, although the higher rate of nasogastric tube reinsertion, a potential source of morbidity, should be taken in account, especially in older patients.

Song et al. focused their attention on the influence of the length of surgical abdominal wound on postoperative recovery. Longer incisions were significantly associated with delay in the first bowel movement, but the effect was not clinically meaningful because this did not change the time of the first passage of flatus, universally recognized marker of postoperative recovery of gut function.

Increasing evidence support the use of indocyanine green fluorescence in oncologic surgery (6, 7), although with a lack of standardization and a quality heterogeneity in several studies (8). Belia et al. provide an overview of the adoption of this technique in the armamentarium of the gastric surgeon. In this field, the equivalence of a totally laparoscopic with the laparoscopic-assisted approach in radical gastrectomy is shown by an interesting retrospective study by Zhong et al.

The deferral of elective procedure during COVID-19 pandemics has created a backlog of millions of surgical procedures, an unexpected challenge for healthcare system worldwide (9). Even in urgent surgery, a shift toward nonoperative management has occurred as revealed by Stavridis et al. in their systematic review on acute cholecystitis management, a similar trend observed in the management of acute appendicitis (10).

The reduced mobility of patients toward high-volume centers during the lockdowns might have suggested alternative surgical strategies to reduce morbidity in low- and medium-volume centers, as proposed by Giuliani et al. in reducing postoperative pancreatic fistula rate after pancreaticoduodenectomy. Albrecht et al. propose the insertion of a negative pressure drainage in the pancreatic duct in the pancreatogastrostomy following pylorus-preserving pancreaticoduodenectomy. The results seem positive, but the small sample (21 patients) and the inherited biases of the design of the study claims caution. On this line, Buonodonno’s team describe the preliminary results of a Hub and Spoke learning program in bariatric surgery in a small region of Italy.

Emerging technologies in gastrointestinal surgery are an exciting and tasty topic. We hope with this number to have partially satiated the hunger of the tablemates.
